# Mid-life predictors of late-life depressive symptoms; determining risk factors spanning two decades in the Women’s Heathy Ageing Project

**DOI:** 10.1186/s40695-020-00050-3

**Published:** 2020-03-04

**Authors:** Katherine E. Campbell, Alexandra Gorelik, Cassandra E. Szoeke, Lorraine Dennerstein

**Affiliations:** 1 School of Psychology, Charles Sturt University, Bathurst, New South Wales Australia; 2 Department of Medicine (RMH), University of Melbourne, Melbourne, Victoria Australia; 3 School of Behavioural and Health Science, Australian Catholic University, Melbourne, Victoria Australia; 4 Department of Psychiatry, University of Melbourne, Melbourne, Victoria Australia

**Keywords:** Late life, Mid-life, Depressive symptoms, CESD, Hassles, Affectometer-2, WHAP

## Abstract

**Background:**

Data available from longitudinal studies of adequate duration to explore midlife risk factors for late life higher depressive symptom scores in women is lacking. This study examines midlife (mean ages 50 years and 60 years) predictors of late life (mean age 70 years) depressive symptom scores to enrich our understanding of the role of changing risk factors across the lifespan.

**Methods:**

This investigation was an assessment of the long-term impact of lifestyle and health variables on depressive symptoms. Data were drawn from an epidemiological prospective study of women’s healthy ageing spanning two decades. Variables included assessment of mood, demographics, physical health, smoking status, attitudes towards ageing and menopause, alcohol consumption and employment. Analysis was conducted to determine the set of strongest predictors assessed in 1992 (mean age 50 years) and in 2002 (mean age 60 years) in relation to higher CESD-SF scores measured in 2012 (mean aged 70 years (*n* = 249)). A cross-sectional analysis determining concurrent associations at mean age 70 years was also conducted.

**Results:**

An increase in positive mood at 50 and 60 years was associated with a 0.3 (95% CI 0.1–0.5) and 0.4 (95%CI 0.1–0.8) point reduction in CESD score at 70 years respectively. An increase in Hassles score at age 50 was associated with a 0.18-point increase in CESD (95% CI 0.01–0.05) 20 years later. However, no relationship was observed between Hassles score at 60 and CESD 10 years later. Analysis of concurrent risk factors demonstrated that bothersome symptom frequency and higher anxiety were associated with higher depressive symptom scores when women were 70 years.

**Conclusion:**

Low levels of positive mood were consistently associated with depressive symptoms scores 10 and 20 years later, suggesting clinical interventions aimed at improving positive affect may be particularly useful across the midlife.

## Background

Mid-life, commonly defined as 45–65 years, represents a time of significant physiological and psychological change for women [1]. With the onset of early postmenopause, severe vasomotor symptoms are more likely to occur [1]. These are also known to be associated with higher reporting of depressive symptoms [2]. In addition to the onset of menopause, other life changes that may be associated with mood fluctuations are common to mid-life women [3]. These include; changes in professional role and work commitments, the onset of physical or health problems, additional responsibility to care for ageing parents, and changes in relationships and sexual functioning [4, 5]. While risk factors associated with higher depressive symptom scores at certain age ranges in women are well documented [5, 6], less is known about the long-term impact of psychological and lifestyle factors experienced during the transition from midlife to late life. Identifying midlife risk factors amenable to behavioural and psychological intervention has the potential to inform the types of clinical intervention used with midlife women who are at risk of later developing depressive symptoms. There is growing consensus that midlife predictors of late life illness are important to mitigate the impact of negative symptoms in old age.

Longitudinal studies spanning the stages of reproductive ageing, with frequent points of assessment, provide the best means for exploring the changing risk factors for women transitioning from midlife to late life. The Women’s Healthy Ageing Project (WHAP), a longitudinal study of women’s healthy ageing, found that risk factors assessed when women were of an average age of 50 years predicted higher depressive symptom scores a decade later [7]. These predictors included: negative attitudes towards ageing; negative attitudes towards menopause; negative mood scores; and prior premenstrual complaints. Another study of longitudinal aging, The Study of Women’s Health Across the Nation (SWAN), examined baseline predictors assessed when women were of mean age 47 years in those who developed Major Depressive Disorder (MDD) by the final assessment point when women were of average age 60 years [8]. These researchers found that the baseline characteristics associated with presence of later MDD included: higher mean trait anxiety, higher mean private self-consciousness, lower mean optimism, higher percentage of lifetime anxiety disorder, two or more lifetime medical conditions, and past use of psychotropic medication [8]. In another study the SWAN team examined the association between physical activity and depression in 2891 women who provided data over 10 years, mean age 42 years at baseline [9]. The findings demonstrated that higher levels of physical activity were associated with lower levels of depressive symptoms persistently over 10 years.

In an examination of factors at the nine-year follow up point in the Penn Ovarian Ageing Study, higher levels of stress at baseline were associated with higher self-reported depressed mood [10]. Across an eight-year follow up, hot flashes, BMI, smoking status, PMS, employment, and marital status were identified as significant risk factors for high CES-D scores [11]. In 2017 Mitchell and Woods published a summary of the factors associated with depressed mood in the Seattle Midlife Women’s Health Study across 13 years of follow up (baseline mean age 41 years) [12]. Individual models, using age as the measure of time, showed that the following variables were associated with higher depressed mood severity: greater perceived stress, having a history of sexual abuse, difficulty getting to sleep, early awakening, and awakening at night [12]. Factors associated with lower depressed mood included: being postmenopausal, exercising more, and having a partner [12].

In this study midlife predictors of late-life depressive symptoms were explored by analysing 20 years of longitudinal prospective data. Depressive symptoms were explored in order to capture more sensitive differences in symptom reporting than could be determined using a categorical diagnosis of presence or absence of Major Depressive Disorder. Using depressive symptom measures rather than clinical diagnosis can also be particularly relevant to this cohort given that older adults are less likely to be diagnosed with a major depressive disorder but are more likely to report more depressive symptoms than their middle-aged counterparts [13]. The purpose of this study was to extend earlier research examining the impact of mid-life variables on depressive symptoms across a 10-year timespan of the WHAP cohort. The duration of the WHAP study now allows for an assessment of the impact of mid-life variables on level of depressive symptoms over a 20-year time frame, providing an opportunity to explore the long-term impact of lifestyle and psychological variables on depressive symptoms. The multiple points of assessment also allow for an examination of the changing risk factors associated with higher depressive symptoms at different life stages. While the previous examination of the WHAP cohort focused on the development of risk factors within the context of reproductive aging, this research examines the variables in the context of chronological ageing, a more relevant context given the age of the cohort.

In this paper we examined lifestyle and health variables associated with early late life higher depressive symptom scores at mean age 70 years. The goal of this analysis was to determine whether risk factors identified when women were aged 50 and 60 remained consistent or represented unique, age specific risk factors. The specific goals of the research were; 1) to assess the association between specific lifestyle and health variables assessed at age 70 in relation to higher depressive symptom scores at age 70 as part of a cross-sectional analysis, 2) to assess the association between specific lifestyle and health variables assessed at age 60 in relation to higher depressive symptom scores at age 70 in order to determine which factors may impact higher depressive symptom scores 10 years later, and, 3) to assess the association between specific lifestyle and health variables assessed at age 50 in relation to higher depressive symptom scores at age 70 in order to determine which factors may impact higher depressive symptom scores 20 years later. Understanding consistent and age specific risk factors predictive of later depressive symptoms would contribute to the understanding of potential modifiable risk factors present during the span of the midlife.

## Method

### Participants

#### Women’s healthy ageing project baseline cohort (1992–2002)

Participants who contributed data to this study were part of the Women’s Healthy Ageing Project (WHAP), an ongoing longitudinal epidemiological study initially known as the Melbourne Women’s Midlife Health Project (MWMHP). The study was originally established to describe Australian women’s experience of the menopausal transition as well as the health and lifestyle factors associated with their experience of the climacteric. As the study continued, additional variables were introduced to explore research questions relevant to the ageing cohort. The women were Australian born Melbourne residents, aged between 45 and 55 years when they were originally contacted by random digit dialling [14]. Sample size at baseline (1992) was 438, with retention remaining high at year 8 of follow-up (88%). A more detailed summary of the cohort and procedure has been described in Dennerstein et al., 2004 [7].

#### Women’s healthy ageing project follow-up cohort (2002–2012)

Assessments were conducted annually between 1991 and 1999, then were readministered in 2002, 2004 and 2012. A more detailed summary of the cohort and procedures, including the 2002 and 2012 assessment points, has been described in Szoeke et al., 2016 [14]. At the 20-year follow-up, 53% of the cohort assessed in 1992 returned for assessment [14, 15]. At each point of contact the study has been approved by the Human Research Ethics Committee of the University of Melbourne. All procedures and ethical standards are in accordance with those outlined by the National Health and Medical Research Council. All women have provided written consent for each time point in which they have participated.

### Measures

#### Centre for Epidemiological Depression Scale (CESD) short form

The CESD-SF is a 10-item, self-report scale used to assess the presence and severity of depressive symptoms in the general population. The measure was introduced into the assessment protocol in 2002. The CESD-SF has been shown to have high reliability (test retest reliability, r = 0.71) and good predictive accuracy when compared to the full 20 item CESD [16]. Scores range from 0 to 30 and have a cut off range of < 10 to categorise normal versus mild to moderate symptoms [17, 18]. A score of 10 or greater is used to categorise an individual as having severe enough levels of depressive symptoms to potentially be diagnosed as clinically depressed [19]. Higher scores on the CESD represent higher levels of depressive symptoms [17].

#### The hospital anxiety and depression scale (HADS)

The HADS is a 14 item self-assessment scale used to assess states of depression and anxiety [20]. Participants rate how much they had been experiencing the emotional state represented by the item during the last week on a scale of 0–3 (with 3 reflecting higher rates). Separate depression (HADS-D) and anxiety (HADS-A) subscales are derived from 7 specific items related to these states. Scores for each of the subscales range from 0 to 21. Sensitivity and specificity for both HADS-A and HADS-D is approximately 0.80, and correlations with other commonly used questionnaires of this nature (such as the Beck Depression Inventory) range between 0.49 to 0.83 [21].

#### Affectometer 2- negative mood, positive mood, wellbeing

The Affectometer 2 is a measure that assesses negative mood, positive mood and wellbeing by asking participants to rate how much they had felt an experience in the past week. Items included 20 adjectives (10 positive and 10 negative) rated on a four-point scale from “most of the time” [3] to “hardly ever” (0) [22]. The mean sum of the scores for the negative adjectives were used to determine the “Negative Mood” score and the positive items were used to determine “Positive Mood”. The “Wellbeing” score was calculated as the difference between the two mood scores [7]. Higher scores represent higher levels of experience for both positive and negative scales [7].

#### The hassles scale

The Hassles Scale is a measure of chronic stress. The scale includes 23 items representing daily “hassles” which were selected and rated as ‘somewhat severe’, ‘moderately severe’ or ‘extremely severe’ to provide a total ‘hassles severity score’ [16]. Daily hassles represent minor negative experiences that occur on a regular basis and are perceived as threatening, irritating, frustrating or distressing [23]. The cumulative nature of these experiences represents ongoing, chronic levels of stress.

#### Attitudes towards ageing

Attitudes towards ageing was assessed using six items rated on a three-point Likert scale to provide a total positive score [24, 25]. A positive response was taken to indicate an overall higher/more positive attitude towards ageing.

#### Attitude to menopause

Seven items consisting of statements related to menopause were rated as ‘strongly agree’; ‘agree’; ‘disagree’ or ‘strongly disagree’ to determine an overall score of attitude to menopause [25–27]. Positive scores were totalled to derive an overall total score, with higher total representing overall higher/more positive attitude towards menopause.

#### Demographic variables

Data relating to age, education (categorized as 0–12 years or greater than 12 years), marital status (categorized as married/defacto or single/divorced) and work status (currently employed or not currently employed) were included in this analysis. The categories used were selected based on those previously used in the published WHAP analyses. For marital status and education sub-categories were collapsed into two larger categories for more meaningful analysis.

#### Lifestyle variables

A number of lifestyle variables were assessed (see [28] for a detailed overview of the variables used in the WHAP). Lifestyle variables used in this analysis included: current smoker (yes/no); exercise for fitness or recreation at least once a week (yes/no); and ‘current drinker’ determined by consumption of alcohol in the last week (yes/no). These groups were categorized based on available data across the study where categories of responses changed over time. The most consistent way to represent alcohol based on different response categories was to identify those that had consumed alcohol in the last week compared to those who had not.

#### Physical activity

A measure of physical activity was introduced in 1993 and has been assessed at each subsequent time point. Participants were asked to rate their level of physical activity. Categories used in this analysis included “often” (4–6 times per week or daily); “regularly” (1–3 times a week), or; “rarely” (<once a month or never).

#### Self-rated health

Participants were asked to rate their health as ‘worse’, ‘the same’ or, ‘better than’ their peers [24]. This was a subjective measure introduced when the study began to compliment other variables associated with attitude towards ageing and attitude towards menopause [7].

#### Bothersome symptoms

A list of 22 symptoms commonly experienced during the climacteric were categorized by participants as either ‘yes this symptom has bothered me’ or ‘no it has not’ [16]. Items included physical symptoms such as: dizzy spells, lack of energy, backaches, difficulty swallowing we well as feeling sad or downhearted. The overall number of bothersome symptoms experienced by the participant provided a total score.

#### Additional health variables

Participants also provided data regarding the presence of a chronic condition (diabetes, asthma, allergies or eczema, hypertension, heart disease, stomach or bowel ulcers, arthritis or rheumatism, cancer, or migraine), current use of prescription medication, and problematic premenstrual changes. Both height and weight were assessed by a trained researcher at each site visit. Participants were asked to remove shoes and heavy clothes prior to this assessment. The height was measured with stadiometer to the closest 0.01 m and weight was measured using digital scale (pre-stabilised) to the nearest 0.1 kg. Body Mass Index BMI (kg/m2) was then calculated using standard equation. Menopausal status was also assessed and categorized using the updated STRAW+ 10 criteria [1]. The date of the final menstrual period (FMP) was recorded by participants in their menstrual diaries and was used to determine menopausal stage.

### Statistical analysis

All continuous variables were tested for normality using Shapiro-Wilk’s test, prior to data analysis. Spearman correlation and Mann-Whitney U-test were used to assess any associations between CESD and participants’ demographic and clinical data. Differences between groups were assessed using either Student T-test or Mann-Whitney U-test for continuous data and either Chi-squared or Fisher’s exact test for categorical data as appropriate. Multivariable gamma regression (with log link) was used to determine the impact of variables assessed in 1992, 2002 and 2012 on primary outcome (CESD-SF score at age 70), while controlling for potential confounders. All models were adjusted for age, BMI, work status, positive affect, marital status and self-rated health as these were consistent between all-time points and were also identified as potential confounders. Certain variables included as predictors were assessed only at a single time-point. Specific variables used at each assessment point are detailed in the corresponding results section. Following gamma regression, the marginal effects were calculated, and results reported in the text refer to the predicted values. All analyses were conducted using SPSS (Statistical Package for Social Science)18 [29] and STATA15 software [30] with *p* < 0.05 considered statistically significant.

## Results

### Concurrent variables associated with higher late life scores (2012)

A total of 249 women (56.8%) of the original cohort assessed in 1992 provided data in 2012. The mean age of the women in this study was 69.8 years (SD 2.57) and the mean score for CESD-SF was 5.20 (SD 4.27). A description of the variables assessed in 2012 is included in Table 1.

**Table 1:** Description of means scores for variables assessed in 2012

2012 Variables	Overall Cohort 2012	Range
Sample Size	249	
Age *Mean (SD)*	69.8(2.6)	64.9–77.3
Affectometer Score		
Negative Mood Score *Mean (SD)*	3.7 (0.34)	1.60–4
Positive Mood Score *Mean (SD)*	1.3 (0.5)	0.9–3.1
Wellbeing Score *Mean (SD)*	2.3 (0.8)	1.1–3.1
Hospital Anxiety Stress Scale		
Anxiety Subscale	4.9 (3.5)	0–17
Depression Subscale	2.4 (2.2)	0–12
Education		
0–12 years	119 (58.6)	
12+ years	84 (41.4)	
Marital Status		
Married/Defacto	154 (67.05)	
Single/Divorced	76 (33.0)	
Physical Activity		
Rarely *n (valid %)*	59 (25.4)	
Sometimes *n (valid %)*	72 (31.1)	
Often *n (valid %)*	101 (43.5)	
Daily Hassles Severity *Mean (SD)*	4.9 (8.5)	
Bothersome Symptoms Frequency *Mean (SD)*	5.7 (4.2)	
Work Status *n (valid %)*		
Not employed	166 (78.7)	
Employed	44 (20.9)	
Self-Rated Health Status *n (valid %)*		
‘Same as/worse than most’	104 (50.5)	
‘Better than most’	91 (19.4)	
Smoking Status		
Smoker	15 (7.1)	
Non-smoker	197 (92.9)	
BMI *Mean (SD)*	28.1 (5.4)	18.3–54.2

*SD* standard deviation

Participants who provided data in 2012, but who were not included in the analysis due to missing items on the CESD (*n* = 29), were significantly more likely to have scored lower on the HADS-D scale (*p* = 0.020) than those included in the analysis. Based on bivariate analysis, smokers had significantly higher CESD score (7 (4–11) vs (4 (2–7) for non-smokers, (*p* = 0.042), and higher scores compared to those employed (5 (2–8) vs 4 (1–7), *p* = 0.039). Women who self-rated their health as “better than most” had significantly lower CESD compared to others (4 (1–6) vs 6 (3–9), *p* = 0.014). The results also show moderate correlation between CESD and negative mood (rho =0.6, *p* < 0.001), positive mood (rho = 0.6–0.5, *p* < 0.001), wellbeing (rho = 0.6, *p* < 0.001), GDS score (rho = 0.6, *p* < 0.001), HADS-A and HADS-D score (rho = 0.6, *p* < 0.001 for both), number of hassles (rho = 0.4, *p* < 0.001) and number of bothersome symptoms (rho = 0.5, *p* < 0.001). Based on bivariate analysis, variables associated with higher CESD scores included: smoking status (*p* = 0.042), self-rated health (*p* = 0.014), work status (*p* = 0.039), negative mood (*p* < 0.001), positive mood (p < 0.001), wellbeing (*p* < 0.001), GDS score (*p* < 0.001), HADS-A score (*p* < 0.001), HADS-D score (*p* < 0.001), number of hassles and number of bothersome symptoms. Refer to Appendix 1 in Table 7 for a summary of these analyses.

However, the results of multivariate analysis showed that bothersome symptom frequency and higher anxiety as measured by the HADS-A only were statistically significantly associated with higher CESD-SF scores (Table 2).

**Table 2:** Multivariate analysis of lifestyle and health variables and CESD scores from 2012

Variable	Beta (95%CI)	*p*-value
Positive Mood Score	−0.24 (− 0.55, 0.87)	0.152
Bothersome Symptoms Frequency	0.04 (0.01, 0.08)	***0.015***
Daily Hassles Severity	0.00 (− 0.01, 0.02)	0.774
HADS-A Total	0.08 (0.03, 0.13)	***0.003***
HADS-D Total	0.03 (− 0.04, 0.15)	0.351

*CI* Confidence Intervals, bold, italicised – significant; Gamma regression was used to derive estimates

Analysis was adjusted for age, education, marital status, smoking status, physical activity, employment, and BMI, none of which showed significance. The results of the analysis indicated that higher scores of bothersome symptoms were associated with a 0.26 (*p* = 0.019) point increase in CESD-SF scores while higher scores on the HADS anxiety measure were associated with a 0.45 (*p* = 0.005) increase in CESD-SF score.

### Ten year predictors of higher late life scores (2002–2012)

A total of 257 women (58.7%) of the original cohort assessed in 1992 provided data in 2002. Of those women 162 had scores for CESD-SF in 2012 in addition to required variables assessed in 2002. The mean age of the women included in the sample used in this study was 59.8 years (SD 2.5) and the mean score for CESD-SF was 6.7 (SD 4.0). A description of characteristics, as well as differences between those included in the 2012 cohort and those who dropped out prior to 2012 are included in Table 3.

**Table 3:** Summary of 2002 cohort and comparison of those who completed 2012 follow-up and those who did not

	Variable 2002	Included in 2012 follow-up assessment	Did not have follow-up data 2012	*p*-value
Sample Size	257	162	95	
Age	59.8 (2.5)	59.8 (2.5)	59.9 (2.5)	^ 0.550
Affectometer Score				
Negative Mood Score	0.3(0.3)	0.3 (0.3)	0.4 (0.3)	^ 0.257
Positive Mood Score	2.2 (0.6)	2.2 (0.6)	2.2 (0.6)	^ 0.783
Wellbeing Score	1.9 (0.8)	1.9 (0.8)	1.8 (0.9)	^ 0.521
CESD-Brief	6.8 (4.1)	6.7 (4.0)	6.8 (4.3)	^ 0.835
Education, n(%)				^∋^0.052
0–12 years	156 (60.7)	91 (56.2)	65 (68.4)	
12+ years	101 (39.3)	71 (43.8)	30 (31.6)	
Marital Status, n(%)				^∋^ 0.943
Married/Defacto	194 (75.8)	123 (75.9)	71 (75.5)	
Single/Divorced	62 (24.2)	39 (24.1)	23 (24.5)	
Physical Activity				^∋^ 0.940
Rarely	45 (17.5)	28 (17.3)	17 (17.8)	
Sometimes	101 (33.3)	65 (40.1)	36 (37.9)	
Often	111 (43.2)	69 (42.6)	42 (44.2)	
Daily Hassles Severity	6.7 (4.1)	6.4 (9.2)	7.1 (9.7)	^ 0.540
Bothersome Symptoms Frequency	7.3 (3.9)	7.1 (3.9)	7.7 (3.9)	^ 0.257
Attitude Towards Ageing	23.1 (2.)	23.3 (2.5)	22.9 (2.4)	^ 0.183
Attitude Towards Menopause	24.4 (3.3)	24.6 (3.3)	24 (3.3)	^ 0.197
Work Status *n(%)*				^∋^ 0.171
Not employed	124 (49.2)	73 (46)	51 (54.8)	
Employed	128 (50.8)	86 (54.1)	42 (45.2)	
Self-Rated Health Status *n(%)*				^∋^ 0.402
‘Same or worse than most’	160 (62.3)	104 (65.2)	56 (35)	
‘Better than most’	97 (37.7)	58 (59.8)	39 (40.2)	
Smoking Status, n(%)				^∋^ 0.406
Smoker	21 (8.2)	15 (9.3)	6 (6.3)	
Non-smoker	236 (91.8)	147 (90.7)	89 (93.7)	
BMI	27.5 (5.6)	28 (5.62)	26.6 (5.4)	^ 0.052

^^^ t-test; Chi-squared test; *p* < 0.05 italicized, bold; *p* < 0.001 included as potential confounder. Results reported as mean (SD), unless otherwise specified

There were no significant differences in lifestyle, health or mood variables between participants who dropped out of the study between 2002 and the 2012 assessment, and those who continued (*n* = 162).

Analysis of associations between individual 2002 factors and 2012 CESD scores demonstrated mild to moderate correlation between 2012 CESD scores and the following variables assessed in 2002: CESD-Brief score (rho = 0.3, *p* = < 0.001); negative mood (rho = 0.3, *p* = < 0.001); positive mood (rho = − 0.4, *p* < 0.001); wellbeing score (rho = 0.4, *p* < 0.001); number of bothersome symptoms (rho = 0.3, *p* = < 0.001) and number of daily hassles (rho = 0.3, *p* < 0.001). However, positive mood remained the only predictor of CESD score 10 years later, while controlling for potential confounders. See Appendix 2 in Table 8 for a summary of the analyses.

The results of the analysis indicated that lower scores on the positive mood scale was a statistically significant predictor of higher CESD scores in 2012 (Table 4). A higher positive mood score resulted in a 2.50 point decrease in CESD total severity (*p* = 0.007).

**Table 4:** Multivariate Analysis of 2002 variables and 2012 CESD scores

Variable (2002)	Beta (95%CI)	*p*-value
Age	0.04 (−0.02, 0.09)	0.173
Positive Mood Score	−0.45 (−0.76, −0.15)	***0.004***
BMI	0.01 (−0.01, 0.04)	0.338
Attitude Toward Ageing	−0.01 (−0.09, 0.07)	0.801
Attitude Toward Menopause	0.02 (− 0.04, 0.08)	0.477
Daily Hassles Severity	0.01 (− 0.00, 0.03)	0.227
Bothersome Symptoms Frequency	0.01 (− 0.03, 0.05)	0.702

*CI* Confidence Intervals; bold, italicised – significant; Gamma regression was used to derive estimates; All models were adjusted for education, work status, positive affect, marital status and self-rated health

### Twenty year predictors of higher late life scores (1992–2012)

A total of 220 women (50.23%) who provided data in 1992 had scores for CESD-SF in 2012 in addition to required variables assessed in 1992 used in the multivariate analysis. The mean age of the women included in the sample used in this study was 49.63 years (SD 2.53) and the mean score for CESD-SF was 5.20 (SD 4.271). A description of characteristics, as well as differences between those included in the 2012 cohort and those who dropped out prior to 2012 is included in Table 5.

**Table 5:** Comparison of 1992 variables for participants who dropped out between 1992 and 2012, and the WHAP cohort remaining in 2012, with signficiant differences between retention cohorts bolded

	1992 Cohort	Included in 2012 follow-up assessment	Did not have follow-up data 2012	*p*-value
Sample Size	438	220	218	
Age *Mean (SD)*	49.6 (2.5)	49.63(2.53)	49.5 (2.4)	^ 0.494
Affectometer Score				
Negative Mood Score *Mean (SD)*	0.5 (0.4)	0.44(0.35)	0.5 (0.4)	***^ 0.029***
Positive Mood Score *Mean (SD)*	2.18 (0.6)	2.18(0.60)	2.2 (0.6)	^ 0.401
Wellbeing Score *Mean (SD)*	1.73 (0.9)	1.74(0.87)	1.7 (0.9)	^ 0.700
Education				
0–12 years	282 (60)	129(58.6)	153 (70.2)	*0.012
12+ years	156 (33.2)	91(41.4)	65 (29.8)	
Marital Status				
Married/Defacto	351 (74.7)	179 (81.4)	172 (79.3)	* 0.581
Single/Divorced	86 (18.3)	45 (20.7)	45 (20.7)	
Daily Hassles Severity *Mean (SD)*	6 (8.24)	5.4 (6.7)	6.6 (9.5)	*^****0.012***
Bothersome Symptoms Frequency *Mean (SD)*	5.8 (3.8)	5.8 (3.8)	5.9 (3.9)	^ 0.504
Attitude Towards Ageing *Mean (SD)*	17.16 (2.67)	15.4 (2)	15.4 (2.1)	^ 0.492
Attitude Towards Menopause *Mean (SD)*	18 (1.95)	18.5 (2.2)	18.2 (2.3)	^ 0.295
Number of Premenstrual Complaints *Mean (SD)*	2.3 (2.6)	1.92 (1.4)	2.57 (3.3)	***^ 0.041***
Self-Reported Physical Disease Current *n (valid %)*	82 (17.4)	45 (20.5)	37 (17.1)	^∋^ 0.362
Work Status *n (valid %)*				
Not employed	106 (22.6)	48 (21.8)	58 (26.9)	^∋^ 0.221
Employed	330 (70.2)	172 (78.2)	158 (73.1)	
Self-Rated Health Status *n (valid %)*				^**∋**^***0.013***
‘Same or worse than most’	211 (49.4)	102 (48.3)	109 (51.7)	
‘Better than most’	216 (50.6)	113 (52.3)	103 (47.7)	
Physical Activity				^∋^ 0.089
Rarely	127 (30)	71 (32.3)	56 (27.5)	
Sometimes	155 (36.6)	86 (39.1)	69 (33.8)	
Often	142 (33.5)	63 (28.6)	79 (38.7)	
Smoking Status				
Smoker	90 (19.1)	37 (16.8)	53 (24.4)	^**∋**^***0.049***
Non-smoker	347 (73.8)	183 (83.2)	164 (75.6)	
BMI *Mean (SD)*	25.9 (4.9)	26 (4.7)	25.8 (5.2)	^ 0.100
Alcohol User *n (valid %)*	282 (60)	149 (67.7)	133 (61.3)	^∋^0.160

^^^ t-test; Chi-squared test; ^*^ Chi-squared test with Fishers’ Exact Test; *p* < 0.05 italicized and bolded; *p* < 0.001 included as potential confounder

Participants who dropped out of the study prior to the 2012 assessment were more likely at the 1992 assessment point to have; a higher number of premenstrual complaints (*p* = 0.041); report more daily hassles (*p* = 0.012); to rate their health as the same or worse than other woman their age (*p* = 0.013); have completed between 13 and 15 years of education *p* = − 0.024); to smoke (*p* = 0.049); and to report lowered mood (*p* = 0.029).

Analysis of association between individual 1992 factors and 2012 CESD scores demonstrated mild to moderate correlation between 2012 CESD scores and the following variables assessed in 1992: current smoking status (6 (3–10) vs 4 (2–7) for non-smokers, *p* = 0.023); negative mood (rho = 0.3, *p* = < 001); positive mood (rho = − 0.3, *p* < 0.001); wellbeing score (rho = − 0.3, *p* < 0.001); number of bothersome symptoms (rho = 0.3, *p* = < 0.001) and number of daily hassles (rho = 0.3, *p* < 0.001). See Appendix 3 in Table 9 for a summary of these analyses.

However, Daily Hassle severity and positive mood remained the only predictors of CESD 20 years later, while controlling for potential confounders as demonstrated in Table 6.

**Table 6:** Multivariate analysis of 2002 variables and 2012 CESD scores

Variable (2002)	Beta (95%CI)	*p*-value
Age	0.05 (− 0.00, 0.09)	0.053
Positive Mood Score	−0.29 (− 0.50, − 0.08)	***0.007***
BMI	0.01 (− 0.01, 0.04)	0.316
Attitude Toward Ageing	−0.01 (− 0.07, 0.05)	0.669
Attitude Toward Menopause	0.02 (− 0.03, 0.07)	0.365
Daily Hassles Severity	0.03 (− 0.01, 0.05)	***0.002***
Bothersome Symptoms Frequency	0.01 (− 0.03, 0.05)	0.662

*CI* Confidence Intervals; bold, italicised – significant; Gamma regression was used to derive estimates; All models were adjusted for education, work status, positive affect, marital status and self-rated health

The results of the analysis indicated that lower scores on the positive mood scale and higher Daily Hassles severity in 1992 were statistically significant predictors of higher CESD scores a decade later in 2012. An increase in Daily Hassles severity score resulted in a 0.18 increase in CESD scores (*p* = 0.004). A higher positive mood score resulted in a 1.55 decrease in CESD total severity (*p* = 0.009).

## Discussion

In this study certain risk factors identified at different age ranges were shown to be more consistently associated with higher reporting of depressive symptoms for women as they transitioned across the midlife and into early late life, age 65 to 77, representing potential specialised targets for early assessment and intervention. Low positive mood scores reported when women were aged 50 and 60 years were associated with higher levels of depressive symptom scores when they were aged 70 years, suggesting that low positive mood may be a consistent midlife risk factor for depressed mood in early late life. Positive mood was more strongly associated with the presence of depressive symptoms than negative mood at ages 50 years and 60 years. Other factors experienced at certain age ranges may also have a long-lasting effect on the risk of depressive symptoms. The number of daily hassles which represents higher experience of chronic stress, reported by women at mean age 50 years, but not 60 years, was associated with higher depressive symptoms at mean age 70. Similarly, cross-sectional analysis conducted when women were at mean age of 70 years demonstrated that women who reported more bothersome symptoms or who had higher anxiety at the time of assessment were more likely to also report higher depressive symptom scores.

The influence of low positive affect on risk of depressive symptoms in this cohort is consistent with other longitudinal studies that have found that low positive affect predicts heightened levels of depression [29–32]. These longitudinal studies include both genders and varying age ranges, including assessment of children [32]. Characteristics associated with positive affect relate not only to happiness but also increased interest, energy and confidence [33]. It has been proposed that these positive characteristics reduce vulnerability to negative events by improving emotional wellbeing and capacity for coping with adversities [34]. It has also been demonstrated that positive mood and emotions broaden attention and cognition and promote flexibility and creative thinking [34]. Positive beliefs about relationship support and personal coping ability have been shown to moderate the effect of life events on suicidality [35]. Several interventions focused on improving Positive Emotionality (PE) have been developed, and preliminary evidence suggests that these types of interventions decrease symptoms amongst currently depressed and anxious individuals [31, 36]. Developing PE in midlife women may improve emotional resilience and reduce the risk of the onset or recurrence of depressive symptoms. Positive mood in midlife may have a longstanding impact on emotional resilience as women enter late life and the findings from this study suggest the need for further examination of positive affect as a long-term protective factor.

The impact of stress on depressive symptoms has been consistently documented, with early life stress being a well-established risk factor for depression [37]. The experience of a stressful life event across adulthood has been shown to be a consistent risk factor [38, 39]. The findings of this study add further support to the potential long-term impact of self-reported chronic stress on depressive symptoms. The bidirectional relationship between stress and depressive symptoms longitudinally needs to be explored further. Those who report a greater level of chronic stress in midlife may have characteristics that make them more vulnerable to experiencing, or reacting more strongly to stressors, similarly to the way that depressogenic characteristics are thought to increase risk of experiencing depressive symptoms [40]. Further research examining the impact of midlife stress on late life depressive symptoms may be useful in identifying the role that chronic stress plays as an ongoing risk factor for increased vulnerability to depressive symptoms.

This study also demonstrated that women who had higher depressive scores at 70 years reported experiencing higher anxiety and more bothersome symptoms concurrently. Within the WHAP cohort a greater number of bothersome symptoms when women were mean age 50 years and 60 years were also identified as predictors for higher CESD-SF scores when women were 60 years old [7]. The presence of bothersome symptoms as a risk factors for women aged 50 years and 60 years may be attributable to different physical vulnerabilities. The final menstrual period occurs, on average, at the age of 51 years [41] and the physical symptoms associated with the early stages of the climacteric may have contributed to higher reports of bothersome symptoms at this time [1]. At mean age 60, physical health can begin to deteriorate, and ongoing physical symptoms associated with the postmenopause can remain. Hot flashes causing discomfort have been shown to be present up to 10 years following the final menstrual period [42]. The combination of these factors may increase the experience of bothersome symptoms at age 60 years.

The impact of physical health on the presence of higher depressive symptoms in late life is commonly reported, and chronic physical health problems have been demonstrated to be amongst the strongest predictors of depressed mood for women in old age [43]. The significant association between number of bothersome physical symptoms and presence of depressive symptoms in this cohort at age 70 years is consistent with findings that chronic disease, decreases in functioning, and chronic pain are all associated with higher rates of depression in older cohorts [44]. The presence and severity of bothersome symptoms appears to be a consistent risk factor for higher ratings of depressive symptom scores in the WHAP cohort, being associated with higher scores at 50 years, 60 years and 70 years [7]. The measure of bothersome symptoms used in this study was developed based on frequently reported physical symptoms associated with the menopause. The items represent general physical concerns associated with that stage of reproductive ageing and it is was an unexpected result to see the degree to which those same physical symptoms were present in the sample when they were 70. The type of physical concerns experienced at menopause does not appear to be unique to that age range. A further analysis of the type of physical symptoms reported at the different age ranges across the mid-life to late life period would be useful to explore menopause specific physical symptoms compared to physical symptoms consistently experienced by women as part of the ageing process.

The association between depressive symptoms and higher anxiety levels demonstrated in this study during the late life period is consistent with research demonstrating comorbidity between these symptoms [45, 46]. Co-occurrence of depression and anxiety has been demonstrated across the life cycle and is associated with a poorer prognosis. This results in greater functional disability and requires more service utilisation than a single disorder [45, 47]. Generalized anxiety and phobias are thought to be the most prevalent disorders in the elderly, and it has been proposed that standard screening for anxiety when depressive symptoms are present may be useful in older populations [37, 46]. Our findings support this and indicate a decade lead time for intervention to reduce burden of disease.

This study utilised data from a group of women across a 20-year time period. While drop-outs were inevitable, the retention rates were relatively high. The longitudinal nature of the study lead to the introduction of new research questions and assessments which resulted in some changes in the way that responses were categorized over time. As a consequence, some variables had inconsistent categories of responses across time points, resulting in the need for broad categories to be used when examining the data across multiple time points. This allowed for general comparison across the two decades but the exact ranges of responses specific to each time point were unable to be utilised. Ideally, additional variables related to answering the current research goal would have been included at the onset of the study, however the establishment of the study over 20 years ago meant that certain relevant variables were not available. In this study clinical diagnosis of depression was not determined and conclusions can only be generalised in relation to depressive symptom scores in educated women from a Western population. The examination of depressive symptom ratings rather than clinical diagnosis in this cohort remains relevant as mild or moderate distress caused by sub-clinical symptoms can cause a significant amount of distress to older adults and needs to be acknowledged [48]. A strength of this study was the inclusion of an anxiety measure as the women aged, given the high occurrence of anxiety symptoms in older adults [46], but it would have been useful to have additional data specific to anxiety from an earlier time point.

Low positive mood scores had a higher association with later reporting of depressive symptoms than higher negative mood scores at both 50 and 60 years. Negative and positive affect have been shown to represent unique dimensions of mood state rather than representing inverse constructs [48, 49]. Future work exploring the strength of positive mood as a protective factor, as opposed to the influence of negative mood as a risk factor may provide an insight into the unique contributions of affect on depressive symptoms in early late life. Further work exploring the long-term impact of chronic stress in more detail may also help to clarify how this type of ongoing stress impacts coping and resilience.

## Conclusion

The current analysis contributes to the understanding of the role of positive affect in association with depressive symptoms in a female cohort representing real world rates of morbidity with data spanning two decades. This work suggests that levels of positive mood in women during the midlife may be a protective factor for late life depressive symptoms and interventions aimed at enhancing positive affect should be explored further. In addition to consistent risk factors, age specific risk factors were also present. The findings from this analysis suggest that at age 50, focusing on chronic stress may be the most helpful point of intervention, while at age 70 an emphasis should be placed on physical symptoms and anxiety. Regardless of the reason for the presence of bothersome symptoms, the severity of these physical symptoms is consistently associated with higher reports of depressive symptoms as women transition from midlife to late life. It may be useful for clinicians to consistently monitor bothersome physical symptoms as a risk factor for depressive symptoms in adult women as they age. Further work would be needed to identify if these age-specific, and consistent, risk factors are evident in other longitudinal cohorts of similar duration.

## Data Availability

The datasets used and/or analysed during the current study are available (subject to consideration from the director of Women’s Healthy Ageing Project) on reasonable request.

## Appendix 1

**Table 7:** Cross-Sectional Association between CESD, lifestyle and health variables in 2012

Variable	rho	CESD Median (IQR)	*p*-value
Age	*0.1*		^***∋***^ 0.107
Affectometer Score			
Negative Mood Score	*0.6*		^**∋**^ ***0.000***
Positive Mood Score	*−0.5*		^**∋**^ ***0.000***
Wellbeing Score	*0.6*		^**∋**^ ***0.000***
Geriatric Depression Scale	*0.6*		^**∋**^ ***0.000***
Hospital Anxiety Stress Scale			
Anxiety Subscale	*0.6*		^**∋**^ ***0.000***
Depression Subscale	*0.6*		^**∋**^ ***0.000***
Daily Hassles Severity	*0.4*		^**∋**^ ***0.000***
Bothersome Symptoms Frequency	*0.5*		^**∋**^ ***0.000***
BMI	0.1		^**∋**^ ***0.000***
Education			^^^0.446
0–12 years		5(2–8)	
12+ years		4(2–7)	
Marital Status			^^^0.734
Married/Defacto		5(4–7)	
Single/Divorced		4(2–7.5)	
Physical Activity			^^^0.441
Rarely		6.1 (2–9)	
Sometime		4.64 (2–7)	
Often		4.97 (2–7)	
Work Status			^***^***^ ***0.039***
Not employed		*5(2–8)*	
Employed		*4(1–7)*	
Self-Rated Health Status			^***∝***^ ***0.014***
‘Same as/worse than most’		*6(2.5–9)*	
‘Better than most’		*4(1.5–6)*	
Smoking Status			^***∝***^ ***0.042***
Smoker		*7(4.5–11)*	
Non-smoker		*4(2–7)*	

^∋^ Spearman Correlation; ^∝^ Mann-Whitney Test; ^ Kruskall-Wallis Test; *p* < 0.05 italicized; *p* < 0.001 included as potential confounder

## Appendix 2

**Table 8:** Associations between 2002 mid-life demographic and clinical factors, and late life CESD scores

Variable (2002)	rho	CESD (2012) Median (IQR)	*p*-value
Age	0.1		^∋^0.111
Affectometer Score			
Negative Mood Score	*0.3*		^***∋***^ ***0.000***
Positive Mood Score	*−0.4*		^***∋***^ ***0.000***
Wellbeing Score	*0.4*		^***∋***^ ***0.000***
CESD-Brief	*0.3*		^***∋***^ ***0.000***
BMI	0.1		^∋^0.318
Daily Hassles Severity	*0.3*		^***∋***^ ***0.000***
Bothersome Symptoms Frequency	*0.3*		^***∋***^ ***0.001***
Attitude Towards Ageing	−0.1		^∋^0.431
Attitude Towards Menopause	−0.1		^∋^0.091
Education			^∝^0.637
0–12 years		4.4(2–7.5)	
12+ years		5(2–7)	
Physical Activity			^^^0.062
Rarely		6.8 (4–8)	
Sometimes		5 (2–7)	
Often		4.9(2–7)	
Work Status			^∝^0.096
Not employed		4(2–7)	
Employed		5(2–9)	
Self-Rated Health Status			^∝^0.050
‘Same as/worse than most’		5.5(3–8)	
‘Better than most’		4(2–7)	
Smoking Status			^∝^0.169
Smoker		*6(3–9)*	
Non-smoker		4(2–7)	
Marital Status			^^^0.367
Married/Defacto		4(2–7)	
Single/Divorced		5.5(3.5–7)	

^∋^ Spearman Correlation; ^∝^ Mann-Whitney Test; ^ Kruskall-Wallis Test; *p* < 0.05 italicized; *p* < 0.001 included as potential confounder

## Appendix 3

**Table 9:** Associations between mid-life demographic and clinical factors in 1992, and late life CESD scores assessed in 2012

Variable (1992)	rho	CESD (2012) Median (IQR)	*p*-value
Age	0.1		^∋^0.089
Affectometer Score			
Negative Mood Score	*0.3*		^***∋***^ ***0.000***
Positive Mood Score	*−0.3*		^***∋***^ ***0.000***
Wellbeing Score	*−0.4*		^***∋***^ ***0.000***
BMI	0.1		^∋^0.162
Daily Hassles Severity	*0.3*		^***∋***^ ***0.000***
Bothersome Symptoms Frequency	*0.3*		^***∋***^ ***0.000***
Attitude Towards Ageing	−0.1		^∋^0.187
Attitude Towards Menopause	−0.1		^∋^0.364
Number of Premenstrual Complaints	0.0		^∋^0.885
Self-Reported Physical Disease Current			^∋^0.093
None reported		4(2–7)	
Physical Disease Reported		6(3–8)	
Education			^∝^0.938
0–12 years		4.9(2–8)	
12+ years		4(2–7)	
Physical Activity			^^^0.350
Rarely		5.8 (2–8)	
Sometimes		4.9 (2–7)	
Often		4.8 (1–7)	
Work Status			^∝^0.559
Not employed		5(3–8)	
Employed		4(2–7.5)	
Self-Rated Health Status			^∝^0.843
‘Same as/worse than most’		4(2–8)	
‘Better than most’		4.8(2–7)	
Smoking Status			^**∝**^ ***0.023***
Smoker		*6(3.5–10)*	
Non-smoker		*4(2–7)*	
Alcohol Use in Last Week			^∝^0.655
Yes		5(2–7)	
No		4(2–9)	
Marital Status			^^^0.247
Married/Defacto		4(2–8)	
Single/Divorced		5(3–7)	

^∋^ Spearman Correlation; ^∝^ Mann-Whitney Test; ^ Kruskall Wallis Test; *p* < 0.05 italicized; *p* < 0.001 included as potential confounder
